# Unveiling Lipid Droplet Transport Dynamics as Biomarkers of Senescence Using Label-Free, Time-Lapse Holotomography

**DOI:** 10.14336/AD.2024.1408

**Published:** 2026-01-19

**Authors:** Amarnath Singam, Nikita Gopakumar, Apoorva Chauhan, Kimberly Ramirez, Jeong Hee Kim, Chandrabali Bhattacharya, Jingchun Chen, Deok-Ho Kim, Seungman Park

**Affiliations:** ^1^Department of Mechanical Engineering, University of Nevada, Las Vegas, NV 89154, USA.; ^2^Interdisciplinary Biomedical Engineering Program, University of Nevada, Las Vegas, NV 89154, USA.; ^3^Department of Mechanical Engineering, Massachusetts Institute of Technology, Cambridge, MA 02139, USA.; ^4^Department of Chemistry and Biochemistry, University of Nevada, Las Vegas, NV 89154, USA.; ^5^Nevada Institute for Personalized Medicine, University of Nevada, Las Vegas, NV 89154, USA.; ^6^School of Integrated Health Sciences, University of Nevada, Las Vegas, NV 89154, USA.; ^7^Interdisciplinary Neuroscience Program, University of Nevada, Las Vegas, NV 89154, USA.; ^8^Department of Biomedical Engineering, Johns Hopkins University School of Medicine, Baltimore, MD 21218, USA.; ^9^Departments of Medicine, Neurology, and Mechanical Engineering, Johns Hopkins University, Baltimore, MD 21205, USA.; ^10^Center for Microphysiological Systems, Johns Hopkins University, Baltimore, MD 21218, USA.; ^11^Institute for NanoBioTechnology, Johns Hopkins University, Baltimore, MD 21218, USA.; ^12^Translational Tissue Engineering Center, Johns Hopkins University School of Medicine, Baltimore, MD 21231, USA.

**Keywords:** senescence, lipid droplet (LD), holotomography, hydrogen peroxide (H_2_O_2_), transport properties, microtubule (MT)

## Abstract

Accumulation and density of lipid droplets (LDs) in cells have been identified as a potential biomarker to detect senescent cells. However, their intracellular dynamic transport and alterations during senescence remain largely unclear. To address this knowledge gap, senescence was induced in human microglia cells using hydrogen peroxide (H_2_O_2_) to investigate both short-term and long-term effects of H_2_O_2_ treatment on LD dynamics. We captured time-lapse holotomograms of LDs using label-free refractive index (RI)-based holotomography and quantified 11 dynamic parameters of LDs through single-particle tracking. These quantified parameters were then compared across healthy cells, short-term H_2_O_2_-treated pre-senescent cells (H_2_O_2_-treated cells), and long-term H_2_O_2_-induced senescent cells (senescent cells). The results revealed that LD dynamics are significantly altered in both H_2_O_2_-treated pre-senescent cells and H_2_O_2_-induced senescent cells, though with differing trends. Healthy cells exhibited higher values in all LD dynamic parameters compared to senescent cells, with the exception of the mean directional change rate, which is lower. In addition, H_2_O_2_-treated cells showed higher values in dynamic parameters such as total displacement, mean straight-line velocity, and confinement ratio compared to healthy and senescent cells, due to the observed linear migration of LDs during H_2_O_2_ treatment. We found that the altered movement of LDs is closely related to H_2_O_2_-induced damage to microtubule networks. These findings suggest that altered LD dynamics, along with associated molecules and pathways, may serve as potential biomarkers for identifying senescent cells, thereby aiding in the development of novel therapeutic targets and senolytics.

## INTRODUCTION

Cells store lipids, including cholesterol ester and triacylglycerol (TAG), within lipid droplets (LDs), utilizing them for energy storage, lipid metabolism, and cellular proliferation [1]. Previous studies have shown that the density and number of LDs are strongly associated with disease progression and pathological states [2-4]. For instance, cancer cells display a higher density of LDs, which correlates with increased aggressiveness [5-7]. Additionally, LDs accumulation is significantly elevated in various disease states across multiple cell types, including neurons and microglia cells (Parkinson’s disease) [8-10], foam cells (atherosclerosis) [11, 12], muscle cells (sarcopenia) [13, 14], T-cells (rheumatoid arthritis) [15], monocytes/macrophages (periodontitis) [16-18], osteoblasts/osteocytes (osteopenia) [19, 20], chondrocytes (osteoarthritis) [21-23], β-cells (type 2 diabetes) [24, 25], and parenchymal hepatocytes (non-alcoholic fatty liver disease) [26].

LDs move dynamically, interacting with other organelles such as mitochondria, peroxisomes, and lysosomes, to carry out diverse functions, including lipid homeostasis, metabolic regulation, and stress response [27-29]. LD motility and transport are closely linked to cellular function and state [1, 30]. Early studies demonstrated that LD velocity or displacement can serve as an indicator of disease progression or severity [1]. Experimental investigations revealed that LD velocity is strongly correlated with tumor aggressiveness through the regulation of PEDF (pigment epithelium-derived factor), V-ATPase (vacuolar-type ATPase), and post-translational modifications of microtubules (MTs) in cancer cells [2]. Another study showed that both LD displacement and mean speed were significantly increased in virus-infected cells, such as those infected by the Zika virus or dsRNA viral mimic [31]. Beyond LD velocity and displacement, additional dynamic parameters have been employed to quantify LD dynamics, including alternating sequences of slow, short-distance movement and fast, long-distance movement, mean square displacement (MSD), directional change, and diffusivity. For example, LD movement within the cytoplasm alternated between slow, short-distance movement and fast, long-distance movement [30]. The findings indicated that the slow movement of LDs tends to be random and diffusive in nature, while rapid movement is more directional, likely facilitated by motor proteins such as kinesin and dynein.

Despite significant research on pathological states, the transport and dynamics of LDs in senescent or aging cells remain underexplored. Studies have demonstrated that aging or senescent cells tend to accumulate a higher number of LDs in the cytoplasm or nuclear region compared to healthy young cells, and these accumulations are closely linked to biological dysfunctions and pathology [32-38]. Consequently, the formation and accumulation of LDs have been reported as a potential marker for detecting and identifying senescent cells [39]. However, to our knowledge, no studies have investigated how LD motility or dynamics are altered during cellular senescence or aging. Changes in the transport properties or dynamics of LDs in senescent cells, as distinct from those in healthy cells, could serve as a valuable, quantifiable measure, complementing LD accumulation as a reliable signature for identifying and monitoring senescence. Given that no single marker is exclusive to senescence, and not all senescent cells exhibit the same characteristics, identifying new markers like LD dynamics would greatly enhance our understanding of cellular senescence and aging [40].

Motivated by this, we hypothesized that LD dynamics undergo significant alterations during cellular senescence, potentially serving as biomarkers for identifying senescent cells. To test this hypothesis, we induced senescence in human microglia cells using hydrogen peroxide (H_2_O_2_) and investigated both the short-term and long-term effects of H_2_O_2_ on LD dynamics. First, we determined the optimal concentration of H_2_O_2_ required to induce cellular senescence while maintaining metabolic activity. Using this optimal concentration, we captured time-lapse holotomograms of LDs in 4D (3D spatial + 1D temporal) via label-free refractive index (RI)-based holotomography during a 1-hour H_2_O_2_ treatment to assess the short-term effects on LD dynamics. To evaluate the long-term effects, we cultured the H_2_O_2_-treated cells in a normal medium for an additional 23 hours before acquiring further time-lapse holotomograms of LDs in senescent cells. Using these time-lapse holotomograms, we quantified 11 parameters to comprehensively measure LD dynamics, including velocity, MSD, directional rate, diffusivity, and more, through single-particle tracking. Finally, we compared these parameters across i) healthy untreated cells, ii) short-term H_2_O_2_-treated cells (i.e., 1-hour H_2_O_2_-treated cells), and iii) long-term H_2_O_2_-induced senescent cells (1-hour H_2_O_2_-treated, followed by 23-hour culturing) to identify any significant differences.

## MATERIALS AND METHODS

### Cell culture and reagents

Human microglia cells (HMC3-CRL-3304, ATCC) were cultured in Minimum Essential Medium (MEM) (Corning, USA) supplemented with 10% fetal bovine serum (FBS) (Cytiva, USA) and 1% Penicillin-Streptomycin (P/S) (Gibco, USA). The cells were maintained in a humidified incubator at 37 °C with 5% CO_2_. Upon reaching 70-80% confluency, the cells were harvested using 0.25% trypsin-EDTA (Gibco, USA) up to 20 passages.

### Induction of cellular senescence using hydrogen peroxide (H_2_O_2_)

The induction of senescence in microglia cells by H_2_O_2_ was performed by replicating the one-step H_2_O_2_ skin fibroblast senescence model reported by Gerasymchuk et al [41]. Specifically, 1 × 10^5^ cells were seeded in a 24-well glass-bottom plate (Cellvis, USA) and allowed to adhere for 16 hours at 37°C with 5% CO_2_. From a stock solution of 9.8 M H_2_O_2_, test concentrations were prepared in supplemented MEM medium at 25, 50, 100, 150, and 200 μM. These solutions were introduced into each well in triplicate and incubated for 1 hour at 37°C with 5% CO_2_. After 1 hour, the H_2_O_2_ solution was replaced with fresh supplemented MEM medium, and the cells were further incubated for an additional 23 hours under the same culture conditions. Following the 24-hour incubation period, the cells were carefully washed with buffer and fixed for immunofluorescence staining. In summary, this study involves three test groups: i) healthy untreated cells, ii) short-term H_2_O_2_-treated cells (i.e., cells treated with H_2_O_2_ for 1 hour), and iii) long-term H_2_O_2_-induced senescent cells (cells treated with H_2_O_2_ for 1 hour followed by 23 hours of culturing in medium).

### MTT assay

The cell viability of microglia exposed to varying concentrations of H_2_O_2_ was assessed using an 3-(4,5-Dimethylthiazol-2-yl)-2,5-Diphenyltetrazolium Bromide (MTT) assay. Briefly, cells were seeded in a 96-well plate at a density of approximately 10,000 cells per well and allowed to attach and grow overnight under standard cell culture conditions. The next day, H_2_O_2_ was introduced into the wells at increasing concentrations of 25, 50, 100, 150, and 200 µM. This treatment was applied for an incubation period of 1 hour, after which the H_2_O_2_-containing medium was replaced with a regular supplemented MEM medium. The cells were then incubated for an additional 23 hours. Upon completion of the incubation period, an MTT (EMD Millipore, Cat# 475989, USA) solution prepared in a MEM-supplemented medium was added to the cells and incubated for 4 hours in the dark. Subsequently, the MTT medium was replaced with 100 µL of DMSO, and absorbance was recorded at 550 nm using a plate reader (Promega GloMax Explorer Multimode Microplate Reader, USA). Relative cell viability was determined by comparing the absorbance of test samples to that of positive control samples using the formula [(absorbance of sample/absorbance of positive control) × 100]. Data are presented as the mean ± standard error (SE) (n=3, representing three independent trials).

### Immunofluorescence staining

The activity of senescence-associated β-galactosidase (SA-β-gal) was analyzed using the CellEvent™ Senescence Green Detection Kit (Invitrogen, Cat# C10851, USA). Briefly, after H_2_O_2_ treatment, microglia cells were washed with buffer and fixed with 4% paraformaldehyde for 12 minutes at room temperature. The fixed cells were subsequently washed with buffer, followed by washes with 0.1% Triton X-100 (ThermoFisher Scientific, USA) and 1% BSA (Sigma-Aldrich, Cat#A9647, USA). The working solution, X-Gal, was prepared according to the manufacturer's instructions and introduced into the wells. The plate was covered and incubated for 2 hours at 37°C in the dark without CO_2_. After incubation, the working solution was removed, and the wells were washed three times with buffer. F-actin was stained using Phalloidin-iFluor 594 (Abcam, Cat# ab176757, UK) by incubating the fixed cells in the working solution (prepared in 1% BSA) for 30 minutes at 37°C. The nuclei of the fixed cells were stained with 1 µg/mL DAPI (Invitrogen, Cat# D1306, USA) for 7 minutes at room temperature and washed three times with buffer. The plate was then imaged using a confocal microscope. Additionally, post H_2_O_2_ treatment, live microglia cells were stained with Tubulin Tracker™ Green (Invitrogen, Cat# T34078, USA) to stain the polymerized MT. Following the product instructions manual, the stock solution of the dye was prepared and further diluted in a 1X live cell imaging buffer (Gibco, USA). The cells were stained with this dye solution by incubating in it for 30 min at 37°C and 5% CO_2_, subsequently the cells were rinsed 3 times with live cell imaging buffer and the nuclei were stained with DAPI prior to image acquisition with confocal microscope. Furthermore, cells not subjected to immunofluorescence stains were used as controls for imaging.

### Lipid droplet staining

The LDs of HMC3 cells were stained with LipidSpot™ 610 (Biotium, Cat# 70069-T, USA) fluorescent dye. Following the manufacturer’s protocol, LipidSpot™ 610 was diluted to 1X in MEM-supplemented medium and introduced to the healthy, and H_2_O_2_ treated cells with further incubation for 30 min at 37°C in the dark. Post incubation, the cells were washed with buffer and then imaged under the Cy5 filter (excitation peak 635 nm) of the holotomographic microscope (3D Cell Explorer-fluo microscope, Nanolive, Tolochenaz, Switzerland) to detect the LDs. Dye-labeled LDs emitted fluorescent signals that coincided with high RI spherical structures observed in the cytoplasmic region of HMC3 cells in the 3D holotomograms.

### Confocal imaging

Confocal images of the fixed immunofluorescent-stained cells were captured using a Nikon A1Rsi Confocal Laser Scanning Microscope (Nikon, Japan) at 20X magnification. The SA-β-gal signal was detected with a 488 nm green filter, the DAPI-stained nuclei were detected with a 405 nm blue filter, and the Phalloidin-iFluor 594-stained F-actin was imaged with a 561 nm red filter. The MT and nucleus of the cells stained with Tubulin Tracker™ Green and DAPI were detected and captured with the aid of 488 nm and 405 nm filters, respectively, at 40X magnifications.

### Quantification of morphological properties

The changes in the morphological properties of healthy cells following H_2_O_2_ treatment were quantified based on fluorescence microscopy images. Briefly, the image pixels were converted to physical length scales, and the intensity, area, perimeter, and shape index (where shape index = 4π × Area/Perimeter^2^) of the DAPI-stained nucleus were measured using the ImageJ software. Similar measurements were performed for the cell body (F-actin stained) and SA-β-gal intensity. Multiple images were captured from three experimental trials, resulting in the analysis of a total of 269 cells, with data averaged for healthy (control) and H_2_O_2_-induced senescent cells. From the MT-stained immunofluorescence images, the intensity of MT staining was measured in control and senescent cells (n=90 cells per biological replicate). Furthermore, the polymerization index of MT in control and senescent cells was measured by calculating the ratio of MT polymers to cell area using Tubeness plugin in ImageJ [42]. Briefly, after running the Tubeness plugin of the binary image, the MT intensity was divided by the cell area to calculate the polymerization index (n=50 cells per biological replicate). All the quantification data for the fluorescence intensity was presented in relation to healthy cells.

### Holotomography imaging and analysis

3D holotomographic imaging of control and senescent microglia cells was conducted to explore the cellular morphology and contents using the holotomographic microscope [43]. Holotomography is a label-free imaging technique based on the principle of RI tomography, which quantifies differences in the RI of cellular components to generate high-resolution 3D images. The method utilizes the scattering and phase shift properties of light as it passes through a sample, with the phase shift being directly proportional to the RI and thickness of the material [44]. More specifically, the holotomographic microscope is a quantitative phase microscope equipped with a low energy 520 nm laser to form an interferometer setup. It emits two beams, an object beam that interacts with the sample and a reference beam. The interaction of these beams forms an interference pattern, or hologram, which encodes the optical properties of the sample. The hologram created from the sample using a rotational scanning mirror is then captured by a CMOS camera and is then computationally reconstructed as a 3D RI distribution, providing detailed insights into the sample’s internal structures and composition. It produces z-stacks of 96 slices with a depth of field of 30 μm. The microscope has the capacity to autofocus and be stable during long-term imaging due to the presence of adjustable mirrors. For imaging, microglia cells were seeded and grown in a µ-Dish 35 mm (Ibidi GmbH, Germany) at 50-70% confluency. At 60X magnification (air objective, NA = 0.8), a cell was focused such that the cell boundary and intracellular organelles like the nucleus, mitochondria, and spherical LDs had clear, distinct, and sharp outlines. Once focused, a 3D image of the cell was captured with calibration of the microscope, followed by a time-lapse acquisition for an hour with one frame every 6 seconds using the built-in STEVE software. To induce cellular senescence, the media in the dish was carefully replaced with media containing 100 µM H_2_O_2_, followed by a time-lapse capture of the same cell under the same parameters. Throughout the acquisitions, the cells were maintained in a humidified chamber at 37°C with 5% CO_2_ on an Okolab stage connected to the microscope. The recorded images were further processed using ImageJ software.

### Single particle tracking for LDs

Single particle tracking of LDs was conducted using the TrackMate plugin in ImageJ [45, 46]. The Laplacian of Gaussian (LOG) spot detection was set to 2 µm (~2.8 pixels), with median filtering and sub-pixel localization enabled. The simple linear assignment problem (LAP) tracker was employed with a maximum linking distance of 3 µm, a maximum gap-closing distance of 3 µm, and a maximum gap-closing frame gap of 2. A filter was applied to include only tracks with seven or more consecutive spots.

### Quantification of LD dynamics

To quantify the LD dynamics, maximum velocity, mean velocity, median velocity, mean straight-line velocity, total displacement, total distance, confinement ratio, linearity of forward progression, mean directional change rate, MSD, and diffusivity were calculated (Supplementary Fig. 1) [47]. The total displacement is defined as the distance between the first and last position of the LD in time. Total distance is the actual traveling distance of an individual LD during the whole time. The mean straight-line velocity is calculated by:

(1) $$\mathrm{Mean}\mathrm{straightline}\mathrm{velocity}=\frac{\mathrm{Total}\mathrm{displacement}}{\mathrm{Track}\mathrm{total}\mathrm{time}}$$

The confinement ratio, also known as the persistence [48] is given by:

(2) $$\mathrm{Confinement}\mathrm{ratio}=\frac{\mathrm{Total}\mathrm{displacement}}{\mathrm{Total}\mathrm{distance}}$$

It describes how far an LD moves from its starting point. This unitless value ranges from 0 to 1. Values close to 0 indicate confined movement, where the particle remains near its starting point. Values close to 1 indicate linear movement, where the particle travels in a straight line with a constant orientation. The linearity of forward progression is as follows:

(3) $$\mathrm{Linearity}\mathrm{of}\mathrm{forward}\mathrm{progession}=\frac{\mathrm{Mean}\mathrm{straightline}\mathrm{velocity}}{\mathrm{Track}\mathrm{mean}\mathrm{speed}}$$

If the track is linear and the time interval between two positions is consistent, then this value equals the confinement ratio. The mean directional change rate is defined as the ratio of the mean directional change to the time interval. The mean directional change, which measures the angle between two successive links and is averaged over all the links in the track, is calculated as follows:

(4) $$\mathrm{Mean}\mathrm{directional}\mathrm{change}=\frac{\sum \theta_{i,i+1}}{N}$$

where N is the number of links and θ_i,i+1_ is the angle between two successive links in radians. The MSD of a single LD describes the average of the squared distances between the LD's start and end positions for all time lags of a certain length τ within one single trajectory [49, 50]:

(5) $$\mathrm{MSD}\left(\tau \right)=<{∆r(\tau )}^{2}>={<[∆r\left(t+\tau \right)-∆r\left(t\right)]}^{2}>$$

where r(t) is the position of the particle at time t, and τ is the lag time between two positions taken by the LD to calculate the displacement (∆r(τ)). The lag time (τ (Δt)) is a function of the acquisition time interval (Δt = 6 s) from frame to frame. More specifically, the MSD of a single LD trajectory is calculated by performing a time average. The mean ± standard error of MSDs over multiple trajectories is estimated through an ensemble average. The diffusivity (D) of the LD is estimated by the formula of normal or Brownian diffusion of LDs in a 2D context [50]:

(6) MSD(τ)=4Dτ

### Statistical Analysis

Statistical analysis was conducted using GraphPad Prism. A two-tailed unpaired Student's t-test with Welch’s correction was employed to compare the means between two groups (between the control and treated groups), while one-way ANOVA was utilized for comparisons among three groups (between the control, H_2_O_2_, and senescence group) (ns: not significant; *, p<0.05; **, p<0.01; ***, p<0.001; ****, p<0.0001). When measurements or analyses were conducted with a small sample size (n < 6), comparisons were performed using non-parametric alternatives such as the Mann-Whitney test for t-tests and the Kruskal-Wallis test followed by Dunn’s post hoc test for one-way ANOVA. All results are presented as mean ± standard error (SE).

## RESULTS

### Morphological and biochemical properties of cells and nuclei during H_2_O_2_-induced cellular senescence

**Figure 1.**
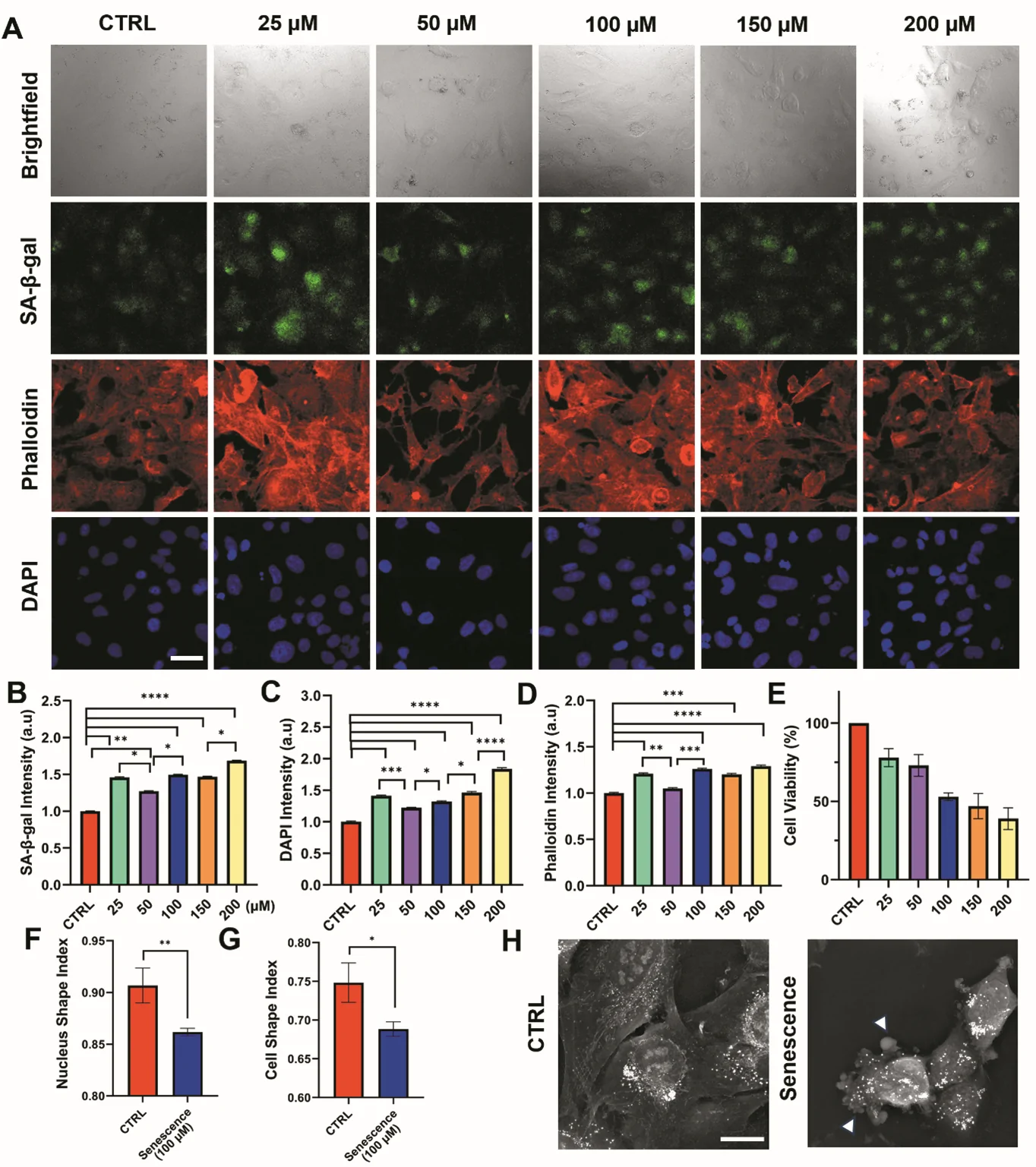
**Hydrogen peroxide (H_2_O_2_) treatment of microglia cells to induce cellular senescence, and quantification of morphological and biochemical features of cells and nuclei**. (**A**) Microglia cells exposed to different concentrations of H_2_O_2_ and visualized in brightfield alongside staining for β-galactosidase activity (senescence-associated-β-galactosidase (SA-β-gal), green), F-actin cytoskeleton (Phalloidin, red), and nuclei (DAPI, blue). Scale bar: 50 µm. (**B**) Normalized fluorescence intensity of SA-β-gal activity in microglia cells at varying concentrations of H_2_O_2_. (**C**) Normalized fluorescence intensity of DAPI staining in microglia cells at varying concentrations of H_2_O_2_. (**D**) Normalized fluorescence intensity of Phalloidin staining in microglia cells at varying concentrations of H_2_O_2_ (~90 cells from n = 3 biological replicates) for panels B-D; a.u.: arbitrary units. (**E**) Cell viability under increasing concentrations of H_2_O_2_ (n=3 independent trials). (**F**) Nuclear shape index of control and senescent cells (~90 cells from n = 3 biological replicates). (**G**) Cell shape index of control and senescent cells (~90 cells from n = 3 biological replicates). (**H**) Refractive index (RI)-based holotomograms of healthy and senescent cells; membrane blebbing indicated by white arrows. Scale bar: 10 μm. Error bars represent mean ± SE. *, p<0.05; **, p<0.01; ***, p<0.001; ****, p<0.0001. CTRL: control. A one-way ANOVA was used to extract p-values in (B)-(D), while a Student's t-test was performed in (F)-(G).

To determine the optimal concentration of H_2_O_2_ for inducing cellular senescence in microglia cells without perturbing their metabolic activities, we exposed healthy cells to five concentrations of H_2_O_2_, ranging from 25 µM to 200 µM (Fig. 1A), for 1 hour. After a 23-hour incubation in normal culture conditions, we observed significant biochemical and morphological changes post-H_2_O_2_ treatment by visualizing β-galactosidase activity (SA-β-gal), the F-actin cytoskeleton (Phalloidin), and the nuclei (DAPI) using confocal fluorescence imaging. The results showed a dose-dependent increase in fluorescence intensity for SA-β-gal, DAPI, and Phalloidin (Fig. 1B-D). The increased SA-β-gal intensity indicated a higher degree of senescence with increasing H_2_O_2_ concentration. Alongside SA-β-gal activity, cell viability was assessed to evaluate H_2_O_2_ toxicity towards microglia using a colorimetric MTT assay (Fig. 1E). Cell viability was 78 ± 5.7 % for 25 µM, 73 ± 6.9 % for 50 µM, 53 ± 2.4 % for 100 µM, 47 ± 8 % for 150 µM and 39 ± 6.9 % for 200 µM. The results showed that cell viability decreased with increasing H_2_O_2_ concentrations. Based on the results, we determined the IC_50_ (half maximal inhibitory concentration) of H_2_O_2_ to be 100 µM. Concentrations above 100 µM were significantly toxic, whereas 25, 50, and 100 µM were relatively safe for inducing senescence without excessive toxicity. Therefore, we used 100 µM H_2_O_2_ for subsequent experiments on short-term H_2_O_2_-treated and long-term H_2_O_2_-induced senescent cells. Using these cell models, we quantified the transport properties of LDs in both H_2_O_2_-treated cells and H_2_O_2_-induced senescent cells. DAPI and F-actin-stained fluorescent images of control and 100 µM H_2_O_2_-induced senescent cells were analyzed to quantify morphological properties such as shape index, perimeter, and area at both the nuclear and cellular levels. A notable decrease in the nuclear shape index was observed in H_2_O_2_-induced senescent cells (0.861 ± 0.003), as compared to 0.906 ± 0.016 in control cells (Fig. 1F). Similarly, the cell shape index significantly decreased in senescent cells (0.688 ± 0.009) compared to control cells (0.748 ± 0.025) (Fig. 1G). In contrast, nuclear area and perimeter were slightly larger in senescent cells, although not statistically significant (807.97 ± 14.73 µm² and 87.43 ± 0.79 µm, respectively), compared to control cells (791.19 ± 13.5 µm² and 85.96 ± 0.72 µm, respectively) (Supplementary Fig. 2A and B). Similarly, cellular area and perimeter in senescent were slightly larger, measuring 175.16 ± 2.61 µm and 2596.45 ± 76.26 µm², respectively, compared to 171.98 ± 2.23 µm and 2458.87 ± 63.32 µm² in control cells (Supplementary Fig. 2C and D). RI-based holotomograms of senescent microglia cells further confirmed these morphological changes, showing altered cell shape along with blebbing of the plasma membrane, as shown in Fig. 1H.

### LDs move toward the nucleus during H_2_O_2_ treatment

**Figure 2.**
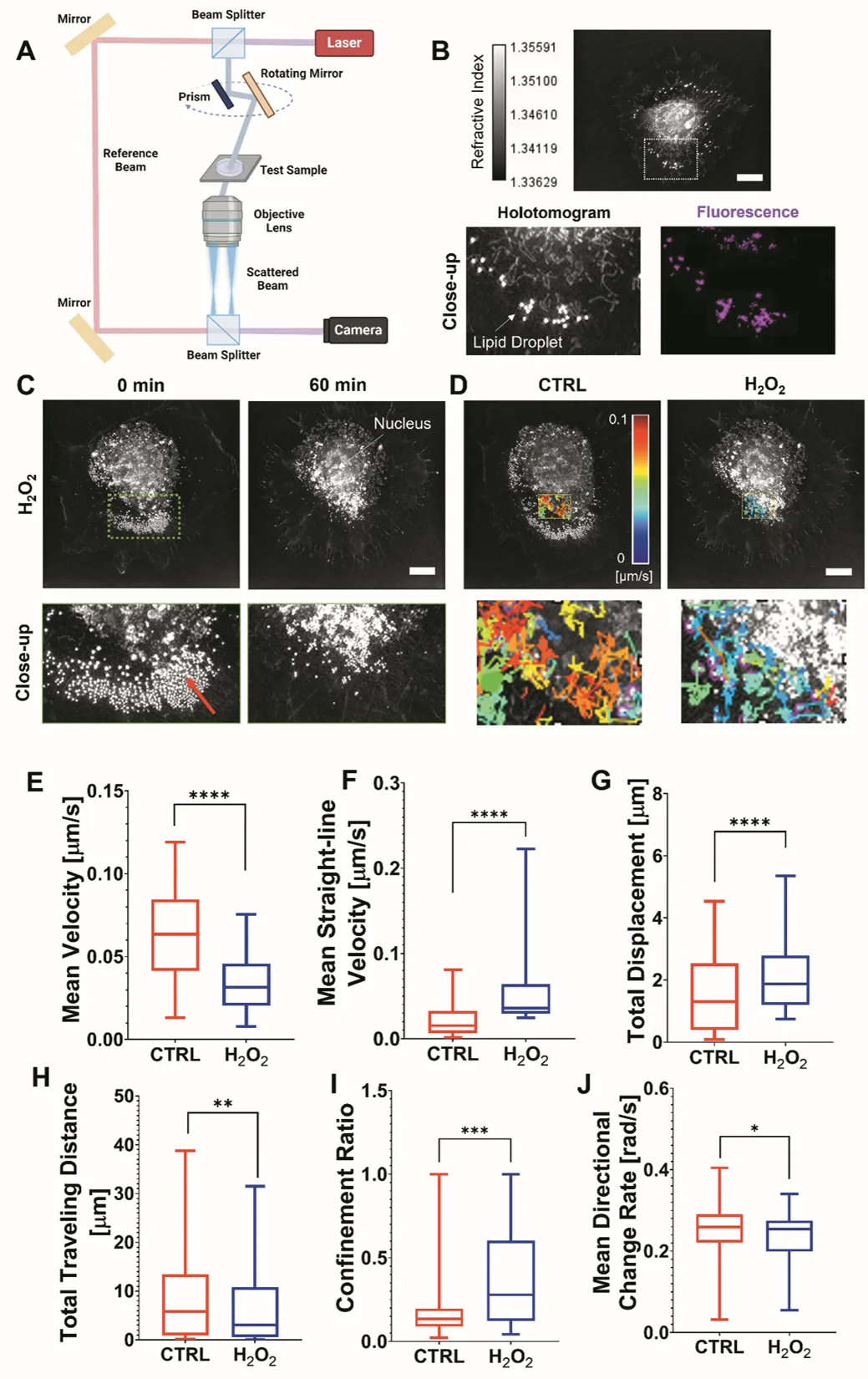
**Dynamics of lipid droplets (LDs) in microglia cells during H_2_O_2_ treatment**. (**A**) Schematic illustration of the holotomography system. (**B**) RI-based holotomogram and fluorescence image of LDs. Enlarged views of the white dashed rectangular regions in the top images provide a detailed visualization of LDs. Scale bar: 10 μm. (**C**) Holotomograms of microglia cells at the initial (0 min) and final state (60 min) during H_2_O_2_ treatment, showing LDs migrating towards the nucleus. The green dashed rectangles highlight close-up images of LDs at the initial and final states. Scale bar: 10 μm. (**D**) Representative images depicting the trajectories of individual LDs in both healthy control and H_2_O_2_-treated cells, with color coding to indicate the mean track velocity of individual LDs. Scale bar: 10 μm. (**E-J**) Quantification of dynamic parameters for LDs in three healthy control cells and three H_2_O_2_-treated cells, including mean velocity (E), mean straight-line velocity (F), total displacement (G), total traveling distance (H), confinement ratio (I), and mean directional change rate (J) (n=380 LDs for control cells and n=382 LDs for H_2_O_2_-treated cells). The box-and-whisker plots display five characteristics of the data: 5th percentile, first quartile, median, third quartile, and 95th percentile. Error bars represent mean ± SE. *, p<0.05; **, p<0.01; ***, p<0.001; ****, p<0.0001. CTRL: control. A Student's t-test was used to extract p-values in (E)-(J).

**Figure 3.**
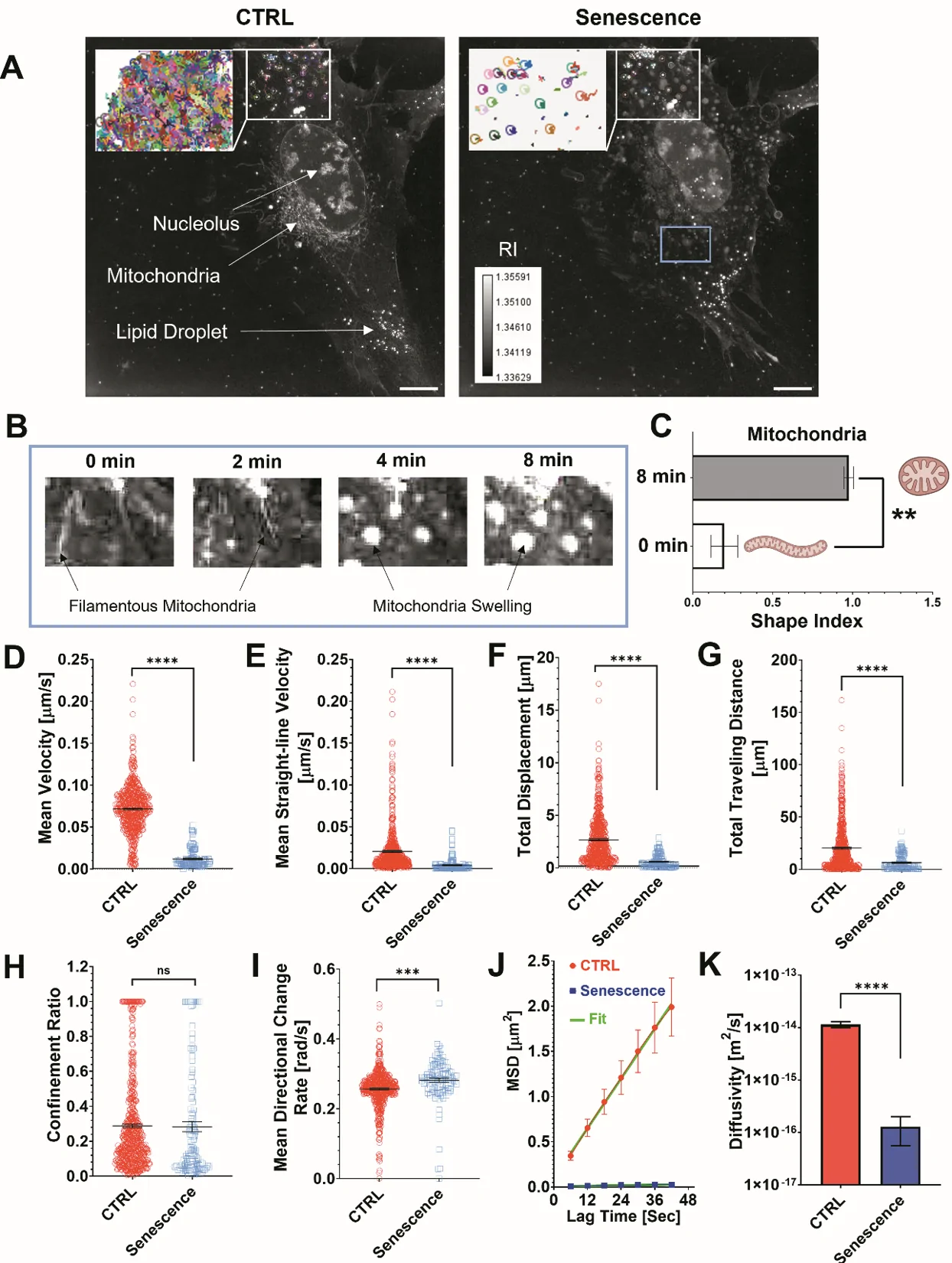
**Dynamics of LDs during H_2_O_2_-induced cellular senescence**. (**A**) RI-based holotomograms of control and senescent cells. Insets show pathlines of individual LDs using a single-particle tracking algorithm. Scale bar: 10 μm (B) Representative images of mitochondrial morphology in senescent cells. (**C**) Quantification of mitochondrial circularity using the shape index. (**D-K**) Various dynamic parameters of LDs in three control cells and three senescent cells, including mean velocity (D), mean straight-line velocity (E), total displacement (F), total traveling distance (G), confinement ratio (H), mean directional change rate (I), mean square displacement (MSD) (J), and diffusivity (K) (n=569 LDs for control cells and n=123 LDs for senescent cells). The y-axis of diffusivity is presented on a logarithmic scale. Error bars represent mean ± SE. ns: not significant; ***, p<0.001; ****, p<0.0001. CTRL: control. A Student's t-test was used to extract p-values in (C)-(I) and (K).

To investigate the effects of H_2_O_2_ on LD dynamics during a 1-hour treatment (i.e., short-term effect), we captured time-lapse holotomograms of both healthy control cells and H_2_O_2_-treated cells using holotomography (Fig. 2A). First, we confirmed that the vesicles observed in the holotomograms were LDs, distinguishing them from other intracellular vesicles, such as lysosomes and stress granules, by performing immunofluorescent staining (Fig. 2B) (The white dashed rectangle in Fig. 2B highlights LDs with RI and fluorescence). The holotomographic images showed that LDs had higher RI values (RI > 1.354) compared to nuclei, mitochondria, and other organelles (1.336 <RI <1.356) (Fig.2C and 3A). The green dashed rectangle in Fig. 2C illustrates LDs in their initial and final states during H_2_O_2_ treatment. The size of LDs (200-300 nm in radius) remained unchanged during H_2_O_2_ treatment (Supplementary Fig. 3). Notably, during H_2_O_2_ treatment, LDs in microglia cells tended to move toward the nucleus in a directed manner (Fig. 2C and Supplementary Movie S1). We observed this phenomenon in other cell types as well, including human foreskin fibroblasts Supplementary (Movie S2). Once near the nucleus, they moved randomly at lower velocities. In contrast, LDs in control cells (Supplementary Movies S3 and S4) continued to move randomly at relatively higher velocities throughout the image acquisition period compared to those in H_2_O_2_-treated cells (Fig. 2D and Supplementary Movie S1 and S2). The yellow rectangle in Fig. 2D shows the pathlines of individual LDs along with their mean track velocity.

Using a Lagrangian-based single-particle tracking method, we quantified the dynamic transport parameters of LDs (Fig. 2E-J, and Supplementary Fig. 4). The mean velocity (Fig. 2E), maximum velocity (Supplementary Fig. 4A), and median velocity (Supplementary Fig. 4B) of individual LDs in the control healthy group were 0.064 ± 0.002 μm/s, 0.166 ± 0.006 μm/s, and 0.058 ± 0.002 μm/s, respectively, which were significantly higher than those of the H_2_O_2_-treated group at 0.037 ± 0.001 μm/s, 0.137 ± 0.005 μm/s, and 0.029 ± 0.001 μm/s, respectively. These results suggest that the LD movement is more active in control cells. Similarly, the total traveling distance of LDs (Fig. 2H) was greater in the control group (10.357 ± 0.787 μm) compared to the H_2_O_2_-treated group (7.841 ± 0.553μm), with diffusivity following a similar trend (Supplementary Fig. 4C). However, the mean straight-line velocity (Fig. 2F) and total displacement (Fig. 2G) of LDs were higher in the H_2_O_2_-treated group (0.054 ± 0.002 μm/s and 2.27 ± 0.075 μm) than in control cells (0.023 ± 0.001 μm and 1.666 ± 0.091 μm, respectively). This is likely related to the fact that LDs followed a straighter path with lower mean directional change during H_2_O_2_ treatment, as supported by the confinement ratio values (0.283 ± 0.02 in the control group and 0.392 ± 0.016 in the H_2_O_2_-treated group) (Fig. 2I), linearity of forward progression (Supplementary Fig. 4D), and mean directional change rate (0.25 ± 0.005 in the control group and 0.235 ± 0.004 in the H_2_O_2_-treated group) (Fig. 2J).

### LD dynamics is markedly impaired in H_2_O_2_-induced senescent cells

To investigate the impact of cellular senescence (i.e., long-term effect) on LD dynamics, we captured time-lapse holograms after a 23-hour incubation in a normal medium following a 1-hour H_2_O_2_ treatment (Fig. 3A and Supplementary Movies S5, S6A and S6B). Similar to the H_2_O_2_-treated cells (i.e., short-term effects), we quantified various dynamic parameters of LDs in H_2_O_2_-induced senescent cells using a Lagrangian-based single-particle tracking method (inset images in Fig. 3A show the pathlines of individual LDs). The images visualizing LD movement revealed that the total trace lengths of LDs in the senescent group were lower compared to those in the control group. Notably, during the senescence process, we observed a drastic change in mitochondrial morphology (blue rectangles in Fig. 3B and 3C). Initially, mitochondria had a filamentous shape at 0 and 2 minutes, but they swelled thereafter (at 4 and 8 minutes), displaying a spherical shape with a higher shape index. Additionally, the size of LDs in senescent cells was approximately twice as large as in control cells (Supplementary Fig. 3).

Quantitative analysis showed that the mean velocity and mean straight-line velocity values of individual LDs were found to be 0.072 ± 0.002 μm/s and 0.021 ± 0.001 μm/s in the control healthy group, which were significantly greater than those in the H_2_O_2_-treated group, at 0.012 ± 0.001 μm/s and 0.004 ± 0.0007 μm/s, respectively (Fig. 3D and 3E). This trend was also observed in the maximum and median velocities (Supplementary Fig. 5A and B). The total displacement of each LD, indicating the distance between its initial and final position, exhibited a 4.79-fold difference between the control group (2.677 ± 0.099 μm) and the H_2_O_2_-treated group (0.587 ± 0.054 μm) (Fig. 3F). The total traveling distance of each LD was 20.30 ± 0.95 μm in the control group, compared to 6.478 ± 0.677 μm in the senescent group, showing a reduction of approximately 3.13 times (Fig. 3G). However, the confinement ratio (Fig. 3H), along with the linearity of forward progression (Supplementary Fig. 5C), which indicates the directionality of LD movement, showed no statistically significant difference between the control group (0.288 ± 0.011) and the senescent group (0.283 ± 0.028). Notably, the mean directional change rate (Fig. 3I) was significantly different, with 0.257 ± 0.002 rad/s in the control group and 0.282 ± 0.006 rad/s in the senescent group. The MSD (Fig. 3J), which indicates the diffusion distance of LDs from their starting positions, was significantly higher in the control group than in the senescent group across all lag times. Consequently, the diffusivity (Fig. 3K) of LDs in the control groups (1.147 × 10^-14^ m²/s) was much greater than in the senescent group (1.28 × 10^-16^ m²/s), exhibiting approximately a 90-fold difference.

### LD dynamics is significantly altered in both short-term H_2_O_2_-treated cells and long-term H_2_O_2_-induced senescent cells

**Figure 4.**
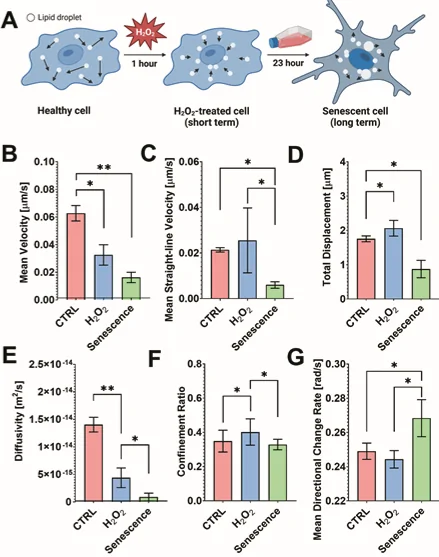
**Comparison of LD dynamics in healthy, H_2_O_2_-treated, and senescence cells**. (**A**) Schematic representation of LD dynamics in different cell states. LDs move randomly in healthy cells. However, after H_2_O_2_ treatment (short-term effect), LDs tend to move toward the nucleus. Once in the perinuclear region, they exhibit random motion at a slower velocity. In senescent cells (long-term effect), enlarged LDs move randomly at a much slower rate compared to the other two groups (healthy and H_2_O_2_-treated). The image was generated by BioRender. (**B-G**) Representative dynamic parameters distinguishing between groups (n=3 biological replicates for each group), including mean velocity (B), mean straight-line velocity (C), total displacement (D), diffusivity (E), confinement ratio (F), and mean directional change rate (G). Error bars represent mean ± SE. *, p<0.05; **, p<0.01. CTRL: control. Significance was determined by Kruskal-Wallis test with Dunn's multiple comparison test in (B)-(G).

Based on the dynamic parameters measured above, we averaged the representative values from at least three cells in each group and identified six key dynamic transport metrics that distinguish among three distinct cell states: control, H_2_O_2_-treated, and senescent cell groups (Fig. 4A). Overall, the mean velocity, mean straight-line velocity and diffusivity in senescent cells were significantly lower than in the other two groups (Fig. 4B, 4C and 4E), indicating a marked decline in dynamic transport efficiency with cellular senescence. Specifically, the mean velocity (Fig. 4B) and mean straight-line velocity (Fig. 4C) in control cells were 0.063 ± 0.006 μm/s and 0.021 ± 0.001 µm/s, respectively. In H_2_O_2_-treated cells, these values were 0.033 ± 0.007 µm/s and 0.026 ± 0.014 µm/s, while in senescent cells, they were 0.016 ± 0.004 µm/s and 0.006 ± 0.001 µm/s. The total displacement (Fig. 4D) and diffusivity (Fig. 4E) in control cells were 1.756 ± 0.084 µm and 0.014 ± 0.001 µm²/s, respectively. In H_2_O_2_-treated cells, these values were 2.063 ± 0.229 µm and 0.004 ± 0.002 µm²/s, while in senescent cells, they were 0.873 ± 0.254 µm and 0.0009 ± 0.0006 µm²/s. The total displacement of LDs in the H_2_O_2_-treated group was greater than in the other groups, consistent with the observations noted above (Fig. 2G). Similarly, for the confinement ratio (Fig. 4F), H_2_O_2_-treated cells (0.402 ± 0.077) exhibited higher values compared to control (0.349 ± 0.064) and senescent cells (0.329 ± 0.031). In contrast, the mean directional change rate (Fig. 4G) was lower in H_2_O_2_-treated cells (0.244 ± 0.005 rad/s) than in control (0.249 ± 0.005 rad/s) and senescent cells (0.268 ± 0.011 rad/s), indicating more directional movement in the control and senescent groups compared to the H_2_O_2_-treated group.

### Altered cytoskeletal structures in senescent cells can impact LD dynamics

**Figure 5.**
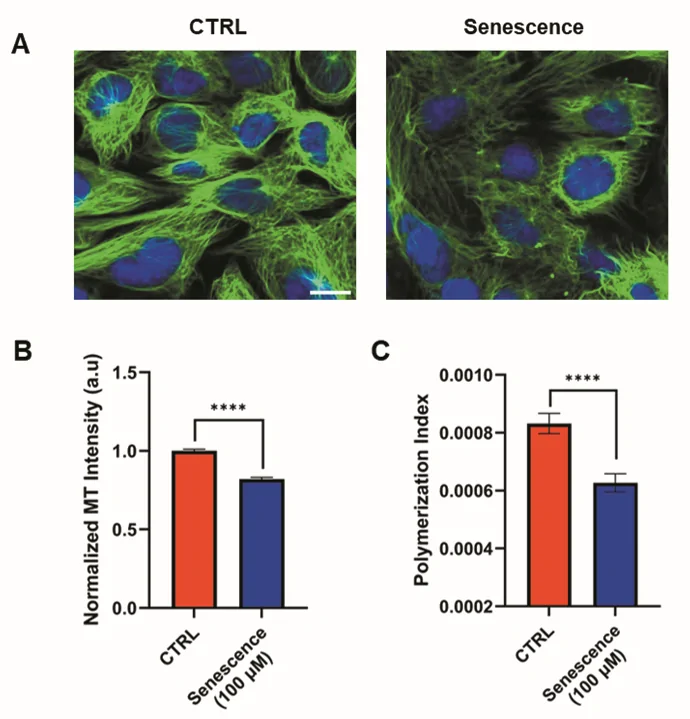
**Investigation of microtubule (MT) dynamics via confocal microscopy (A) Fluorescence images of MT stained with Tubulin Tracker in healthy and senescent cells**. Scale bar: 20 μm. (**B**) Normalized fluorescence intensity of MTs in healthy and senescent microglia cells (90 cells from n = 3 biological replicates). a.u.: arbitrary unit. (**C**) Polymerization index of MTs in healthy and senescent microglia cells (50 cells from n = 3 biological replicates). Error bars represent mean ± SE. ****, p<0.0001. CTRL: control. A Student's t-test was used to extract p-values in (B)-(C).

Intracellular LD trafficking is predominantly mediated by the intricate network of cytoskeletal fibers, particularly actin filaments and MTs, which are orchestrated by cytoskeleton-associated motor proteins such as myosins, kinesins, and dyneins [51, 52]. Despite extensive research, the specific cytoskeletal components and molecular adaptors that regulate LD motility remain largely unidentified, leaving a significant gap in our understanding of the mechanisms underlying LD motility. MTs, in particular, play a crucial role in the transport and fusion of LDs [53]. Thus, to determine whether the impaired dynamics and movement of LDs in senescent cells are linked to the integrity of cytoskeletal structures, we visualized MT structures and arrangements using immunofluorescence (Fig. 5A and Supplementary Fig. 6).

From the fluorescence images, we quantified MT fluorescence intensity (Fig. 5B) and the polymerization index (Fig. 5C). The results showed a significant reduction in both fluorescence intensity and MT polymerization in senescent cells, indicating that MT structural integrity and organization are substantially compromised during cellular senescence. Notably, some MTs in senescent cells appeared disconnected and fragmented, in contrast to the continuous and organized MTs observed in healthy cells (Fig. 5A). Given the critical role of MTs in facilitating LD transport, their disruption likely impairs the movement of LDs, as observed in this study, thereby affecting cellular function and homeostasis [54, 55].

## DISCUSSION

In our investigation, a significant alteration in mitochondrial morphology was observed, transitioning from a filamentous to a spherical configuration during cellular senescence (Fig. 3B and 3C). Despite extensive research, the exact mechanisms underlying mitochondrial swelling remain partially understood [54]. The changes in mitochondrial morphology in H_2_O_2_-induced senescent cells can be attributed to several interconnected biochemical and structural disruptions. One prominent hypothesis suggests that reactive oxygen species (ROS) are involved in the opening of the permeability transition pore (PTP) [55]. ROS are known to oxidize proteins and lipids, disrupting the redox balance within mitochondria. These oxidative modifications may interfere with ion gradients across the mitochondrial membrane, facilitating aberrant PTP opening and subsequent mitochondrial swelling [56]. In addition, H_2_O_2_ exposure can lead to hyperpolarization of the mitochondrial membrane potential and altered mitochondrial activity, which can disrupt the normal function of the electron transport chain [57]. Furthermore, H_2_O_2_ exposure may result in decreased ATP levels and elevated intracellular calcium concentrations, both of which are critical for maintaining mitochondrial integrity and function. Fluctuating oxidative stress specifically induces large-amplitude calcium fluctuations, further exacerbating mitochondrial dysfunction. However, further research is required to fully understand the complexities of mitochondrial swelling and its implications in senescence and aging [58].

A notable change in cellular shape index was observed between healthy control and senescent cells (Fig. 1G). Senescent cells exhibited a reduced shape index, indicating a more irregular and non-circular morphology. This irregularity is likely due to the formation of membrane blebs prominently visible in the senescent cells (Fig. 1H). We also found a slight increase in cellular and nuclear perimeter and area during H_2_O_2_-induced cellular senescence (Supplementary Fig. 2 and 7). One potential reason for the increase in nucleus size during senescence is the formation of senescence-associated heterochromatin foci (SAHF) [59] as indicated by the elevated DAPI intensity - representing chromatin condensation - during cellular senescence (Fig. 1C). SAHF are densely packed heterochromatic regions formed in the nucleus of senescent cells, characterized by chromatin compaction and the sequestration of factors involved in gene expression [60, 61]. As chromatin becomes more compacted and organized into SAHF, it may occupy more space within the nucleus, leading to an increase in nuclear size. Additionally, nuclear membrane proteins like lamin A and B are altered during aging/senescence with modifications of the nuclear envelope (NE) and nuclear pores, resulting in an enlarged nucleus. This also results in the leakage of DNA fragments from the nucleus into the cytoplasmic region, leading to the activation of the cGAS-STING pathway of the immune system, thereby stimulating the senescence-associated secretory phenotype (SASP) [61]. In holotomographic imaging, we noticed a cloudy nucleus in senescent cells displaying increased RI intensity in the nucleoplasm of senescent cells, presumably due to the elevated SAHF (Supplementary Movie 7 and Supplementary Figure 8), which could occur due to high protein turnover in oxidative stress conditions. In human fibroblasts, the nuclear enzymatic machinery was found to be affected by H_2_O_2_, causing oxidation and accumulation of proteins [62].

In parallel with the increase in nuclear size, the cell body size also expanded in the senescent state (Supplementary Fig. 2), likely due to the continuous accumulation of biomass in the absence of cell division—a consequence of the loosely coupled nature of cell growth and division [63]. More specifically, damage-induced cell cycle checkpoints effectively halt cell division, yet the biosynthesis of macromolecules continues unimpeded, driving sustained cellular growth. As a result, these cells experience hypertrophy, increasing size without a corresponding rise in DNA content.

To date, only a limited number of studies have explored the relationship between LD behaviors and cellular senescence or aging. Most of these investigations have primarily focused on the correlation between LD static behaviors, including accumulation or quantity and cellular senescence. However, to our knowledge, no prior study has examined how LD dynamic behaviors are altered during the process of cellular senescence or aging. In this study, we presented the first potential evidence that LD migration patterns and motility undergo significant changes during pre-senescence and senescence, suggesting their potential as biomarkers for cellular senescence using label-free, RI-based holotomography.

Upon exposure to H_2_O_2_ (i.e., the short-term effect of H_2_O_2_), LDs exhibit dynamic behavior, migrating towards and aggregating in the perinuclear region. This phenomenon can be explained by a concurrent reorganization of the cytoskeleton, characterized by a pronounced concentration of tubulin around the nucleus compared to the cell periphery [43]. Our immunofluorescence images revealed that MTs display increased alignment toward the nucleus during H_2_O_2_ treatment, with LDs exhibiting directional movement along them (Supplementary Fig. 6B) compared to the healthy control group (Supplementary Fig. 6A). Such altered cytoskeletal architectures play a key role in regulating the observed redistribution of LDs, providing a compelling avenue for further research into the mechanistic underpinnings and functional implications of this response to oxidative stress. Another plausible explanation of LD movement toward the nucleus is that oxidative stress, induced by H_2_O_2_, can lead to significant cellular and organelle changes, including the formation of multinucleated cells and centrosome hyper-amplification, which are indicative of disrupted cell cycle progression and mitotic defects [64]. This stress also triggers nucleolar segregation, a process that can be mitigated by heat shock proteins like Hsp70, which translocate to the nucleus and nucleolus to exert protective effects [65]. This shift in transcript localization under stress conditions suggests a reorganization of cellular components, including LDs, to support nuclear functions and maintain cellular homeostasis [66]. The movement of LDs toward the nucleus could thus be a strategic cellular adaptation to ensure that essential lipids and proteins are readily available to the nucleus for repair and survival processes during oxidative stress. This reorganization helps the cell to efficiently manage the increased demand for protective and repair mechanisms, highlighting the intricate interplay between cellular structures and stress responses.

In senescent cells, indicative of the long-term effects of H_2_O_2_ exposure, we observed a marked reduction in the mean and median velocities, straight-line velocities, total displacement, and overall traveling distance of LDs. Notably, while the mean directional change rates decreased in H_2_O_2_-treated cells, they drastically increased in senescent cells (Fig. 4G), indicating the more directional movement of LDs during H_2_O_2_ treatment with smaller directional changes compared to control and senescent cells. Further characterization of LD motility through MSD and diffusivity analyses revealed a significant decrease in these parameters in senescent cells relative to control cells. This reduction may be attributable to the enlarged size of LDs in senescent cells, which are approximately twice as large as those in control cells (Supplementary Fig. 3), resulting in slower movement. Another plausible explanation behind the slower motion of LDs in cellular senescence involves the dysregulation and damage of transport cytoskeletons such as MTs or related proteins, as observed in this study (Fig. 5A), which are known to serve as conduits for LDs and facilitate the transport of cargo proteins within the cytoplasm as mentioned above [67, 68]. Previous studies have demonstrated that LDs move along the cellular network of MTs or F-actin via dynamic motor proteins, including myosin, kinesin, and dynein [52, 69-71]. These proteins are essential for connecting and transporting LDs along MTs or F-actin. Their disruption or dysfunction during senescence could severely impair LD motility, leading to the LDs moving very slowly with random motions. Building on this, we visualized and quantified the structural integrity of both MTs and F-actin. Intriguingly, our findings revealed a significant decrease in MT intensity and polymerization during senescence (Fig. 5B and 5C), leading to impaired movement of LDs along disrupted MT (Supplementary Fig. 6C). The polymerization process of MTs or MT-associate motor proteins such as kinesin or dynein may be disrupted during cellular senescence or aging, leading to aberrant LD dynamics. Prior studies have shown that oxidative stress can induce MT depolymerization and alter mitochondrial mass in cells [72, 73]. In another study, MT disassembly led to the dislocation of inositol lipids from the cell membrane during blebbing in *Dictyostelium discoideum* cell [74].

In contrast to MTs, the F-actin cytoskeletal structure exhibited an increased amount of F-actin in senescent cells compared to control cells (Fig. 1D). Moreover, in senescent cells, the F-actin filaments were significantly thicker and longer, forming highly oriented, bundle-like structures, while F-actin filaments in healthy control cells were loosely arranged with more homogeneous features (Supplementary Fig. 9). Previous studies have shown that a lower pH environment promotes the formation of more bundle-like F-actin structures [75]. Consequently, it is suggested that the addition of H_2_O_2_ decreases the pH, leading to alterations in the arrangement and structure of F-actin. These results support the hypothesis that MT degradation, rather than F-actin disruption, may play a primary role in the impaired and altered LD transport observed during cellular senescence. Further studies are warranted to elucidate the alterations in the architecture of the MT network in aging or senescent cells.

Numerous potential senescence markers have been proposed based on morphological, biochemical, and epigenomic changes, including SA-β-gal, lipofuscin, p16, p21, and various proinflammatory cytokines and chemokines [76-80]. However, a universally robust marker for detecting cellular senescence has not been established due to the significant variability in senescence-related features and markers, which are highly dependent on cell type and the nature of the inducing stressor. For example, extrinsic stress-induced senescent cells typically exhibit strong SASP expression, whereas replicative senescence results in weaker SASP expression [81]. Our experimental results further revealed this heterogeneity; senescent fibroblasts treated with H_2_O_2_ exhibited numerous vacuoles, while senescent microglia displayed pronounced blebbing and irregular cell boundaries (data not shown). While SA-β-gal is widely regarded as the most robust senescence marker across various cell types and stressors, it is not universally reliable, as certain cell types do not exhibit strong SA-β-gal activity [82]. Moreover, assessing SA-β-gal activity requires staining dyes that can potentially damage cells and organelles, distorting their dynamics and compromising cellular integrity [83]. Therefore, additional solid markers at the cellular, organelle, and molecular level should be explored to enable more accurate identification of senescent cells.

## Conclusion

In this study, we utilized label-free 3D holotomography, a technique that enables long-term monitoring and imaging of cellular changes without using fluorescent probes. Using this innovative approach, we demonstrated, for the first time, that the transport properties and kinetics of LDs could serve as a promising biomarker for cellular senescence. Specifically, dynamic parameters such as LD mean velocity, mean straight-line velocity, total displacement, confinement ratio, diffusivity, and mean directional change rate were significantly altered in senescence cells. These findings could serve as a foundation for identifying new therapeutic targets and developing senolytics to reverse cellular senescence or aging.

## Supplementary Materials

The Supplementary data can be found online at: www.aginganddisease.org/EN/10.14336/AD.2024.1408.

## Data Availability

The data that support the ﬁndings of this study are available from the corresponding author upon reasonable request.
