# Supplementary material for: Unveiling Lipid Droplet Transport Dynamics as Biomarkers of Senescence Using Label-Free, Time-Lapse Holotomography
Source: Aging Dis. 2025 Jan 19;17(1):549–65. doi: 10.14336/AD.2024.1408 (PMC12727127; doi:10.14336/AD.2024.1408)
Supplement: Supplementary file 1 — The Supplementary data can be found online at: www.aginganddisease.org/EN/10.14336/AD.2024.1408. [file AD-17-1-549-s.pdf]

SUPPLEMENTARY DATA

Unveiling Lipid Droplet Transport Dynamics as Biomarkers of Senescence Using Label-Free, Time-Lapse Holotomography

Amarnath Singam, Nikita Gopakumar, Apoorva Chauhan, Kimberly Ramirez, Jeong Hee Kim, Chandrabali Bhattacharya, Jingchun Chen, Deok-Ho Kim, Seungman Park

© 2024. Singam S. et al. Published online at http://www.aginganddisease.org/EN/10.14336/AD.2024.1408

SUPPLEMENTARY DATA

SUPPLEMENTARY MOVIES

Movie S1. Time-lapse holotomogram of H_2O_2-treated microglia cells (short-term effect, 1 hour). Scale bar: 10 µm.
Movie S2. Time-lapse holotomogram of H_2O_2-treated fibroblasts (short-term effect, 30 minutes). Scale bar: 10 µm
Movie S3. Time-lapse holotomogram of healthy microglia cells (short-term effect, 1 hour). Scale bar: 10 µm
Movie S4. Time-lapse holotomogram of healthy fibroblasts (short-term effect, 30 minutes). Scale bar: 10 µm
Movie S5. Time-lapse holotomogram of healthy microglia cells (long-term effect, 1 hour). Scale bar: 10 µm
Movie S6A. Time-lapse holotomogram of H_2O_2-induced senescent cells (long-term effect, 1 hour) - Example 1. Scale bar: 10 µm
Movie S6B. Time-lapse holotomogram of H_2O_2-induced senescent cells (long-term effect, 1 hour) - Example 2. Scale bar: 10 µm
Movie S7. Nucleoplasm surface intensity of control and H_2O_2-induced senescent cells. Scale bar: 10 µm

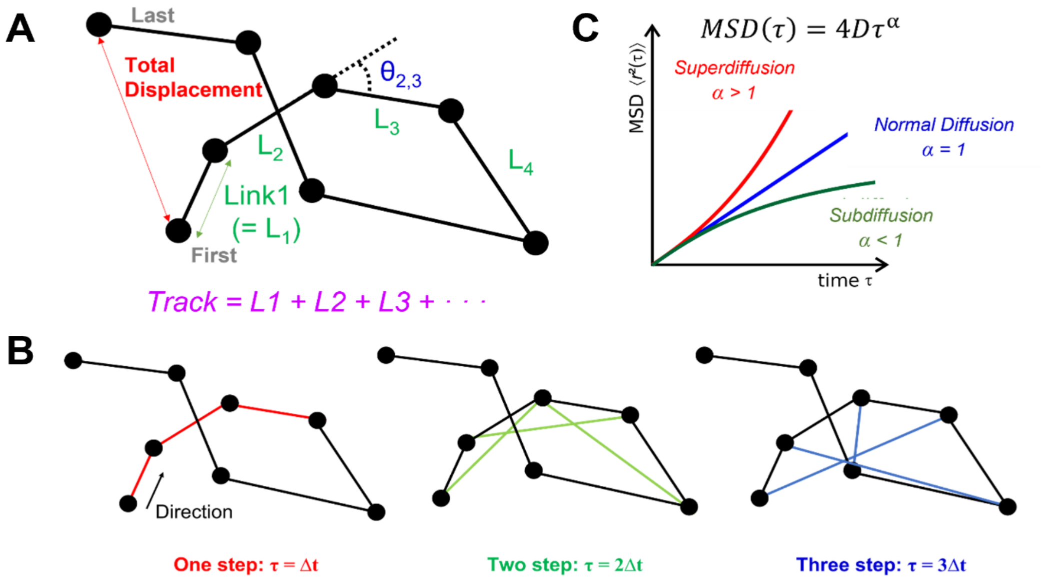

Supplementary Figure 1. Graphical Schematic of Lipid Droplet (LD) Dynamic Random Motion. A) A schematic representation of a trajectory between successive frames (black circles on the trajectory) with an acquisition time step (Δt). This is used for quantifying LD dynamic parameters such as total displacement, velocity, traveling distance, and more. B) Examples of lag time (τ) estimation as a function of Δt: τ = Δt (red), τ = 2Δt (green), τ = 3Δt (blue), and so on. C) MSD plot, averaging all steps within the trajectory for each τ. Normal diffusion is indicated by α = 1, while anomalous diffusion is characterized by α < 1 (subdiffusion) or α > 1 (superdiffusion).

© 2024. Singam S. et al. Published online at http://www.aginganddisease.org/EN/10.14336/AD.2024.1408

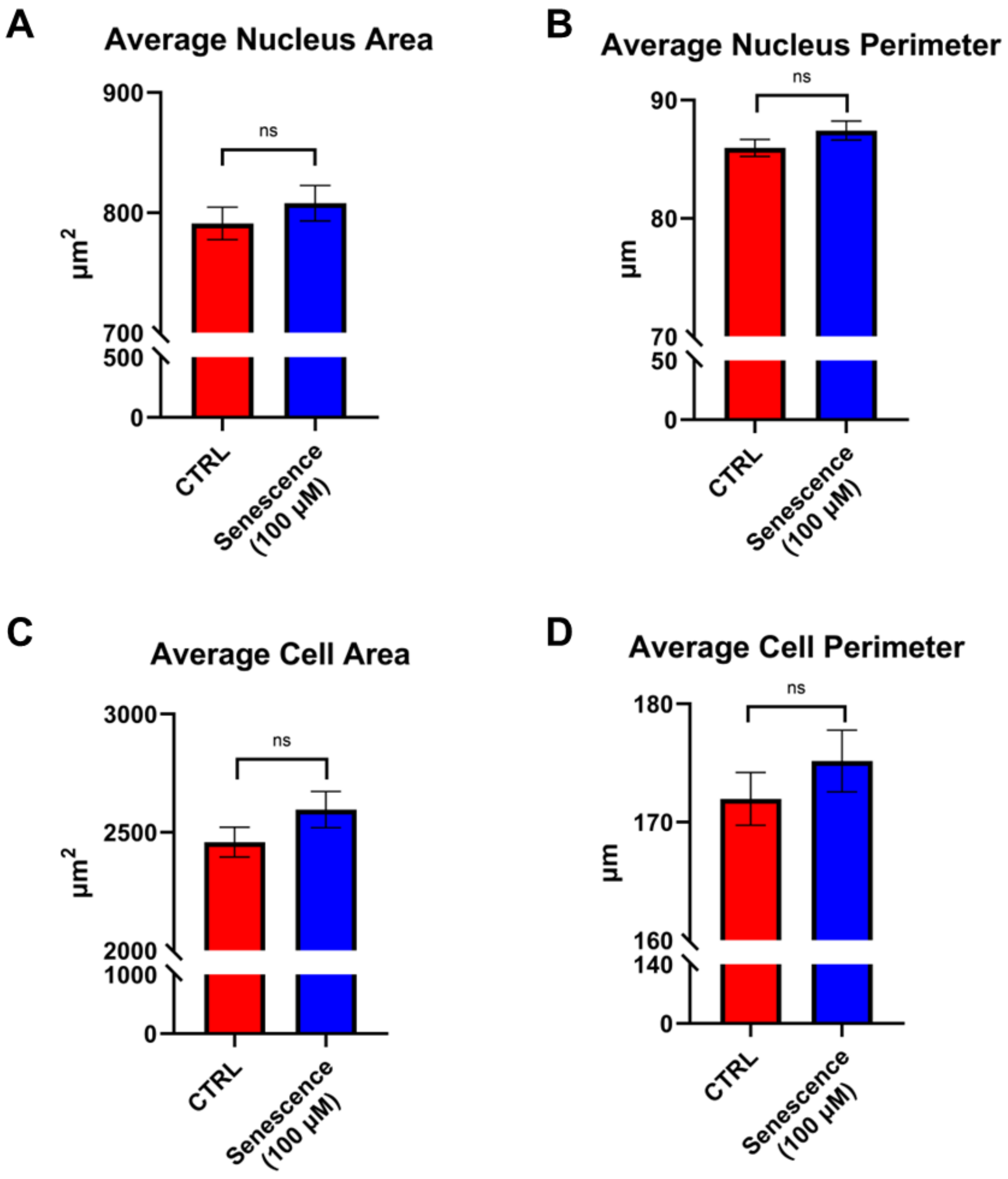

Supplementary Figure 2. Morphological properties in H_2O_2-induced cellular senescence: A) nuclear area, B) nuclear perimeter, C) cellular area, and D) cellular perimeter. Both the area and perimeter of nuclei and cells tend to increase during cellular senescence, although the differences are not statistically significant (~90 cells from n=3 biological replicates). ns: not significant.

© 2024. Singam S. et al. Published online at http://www.aginganddisease.org/EN/10.14336/AD.2024.1408

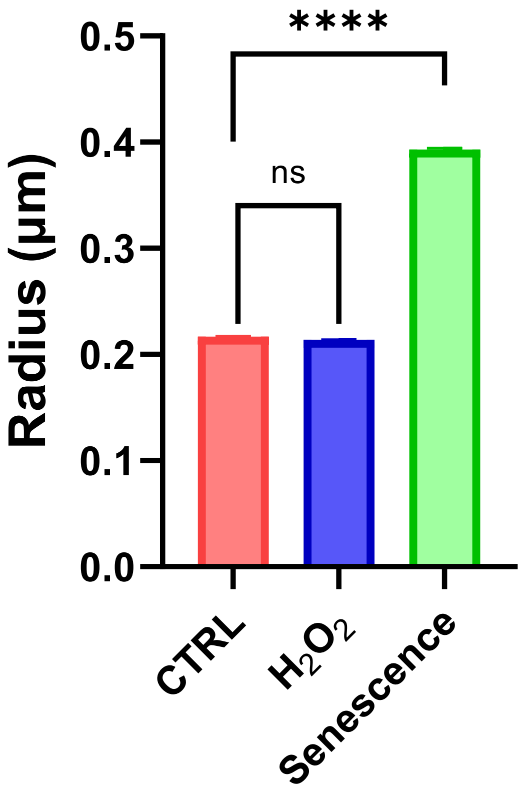

Supplementary Figure 3. Size of lipid droplets (LDs) in control, H_2O_2-treated, and senescence cells (n=3 biological replicates). ns: not significant; ****, p<0.0001.

© 2024. Singam S. et al. Published online at http://www.aginganddisease.org/EN/10.14336/AD.2024.1408

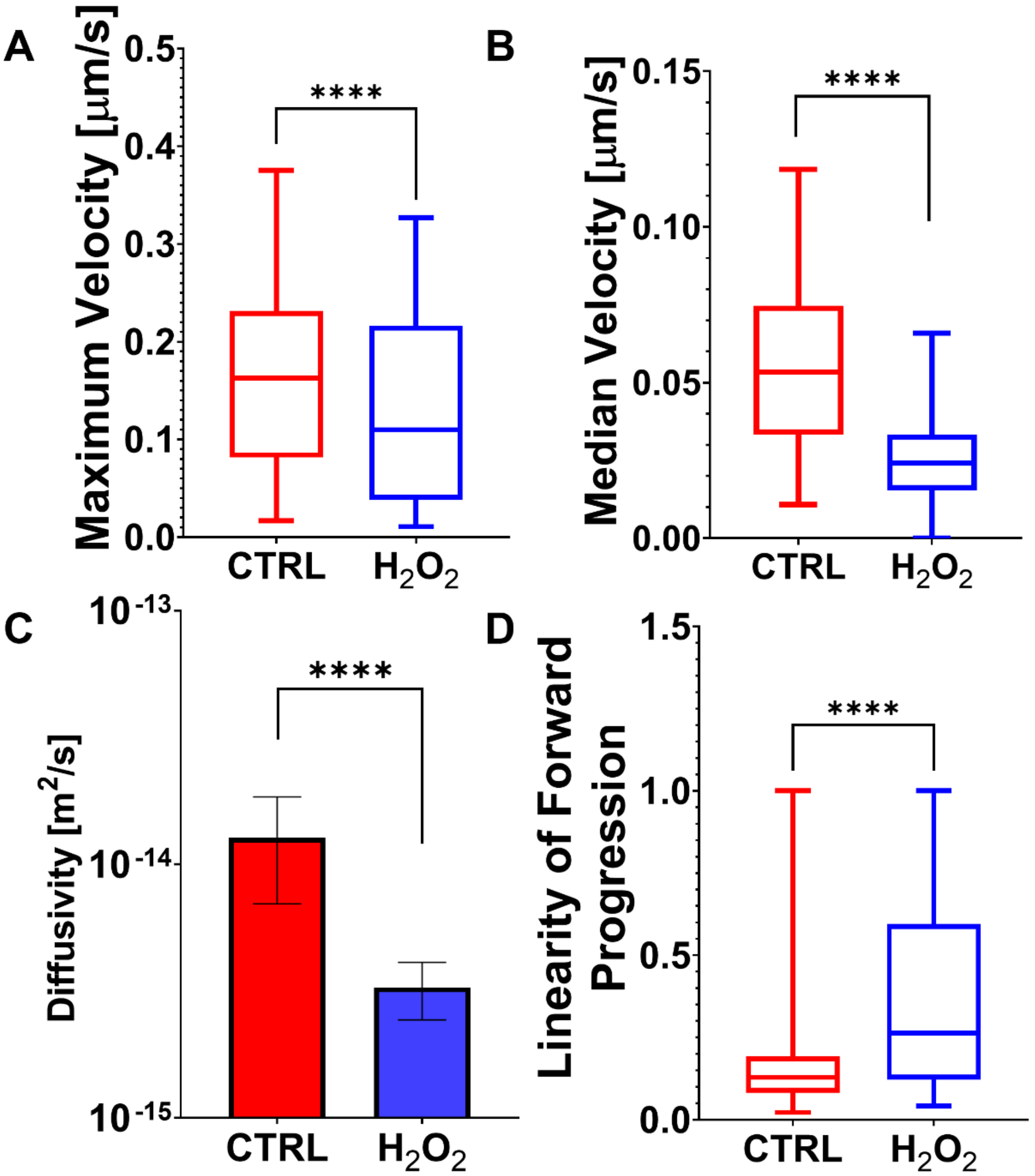

Figure S4. Dynamics transport parameters of LDs in control and H_2O_2-treated cells: A) maximum velocity, B) median velocity, C) diffusivity, and D) linearity of forward progression (n=380 LDs for control cells and n=382 LDs for H_2O_2-treated cells). ****, $p<0.0001$

© 2024. Singam S. et al. Published online at http://www.aginganddisease.org/EN/10.14336/AD.2024.1408

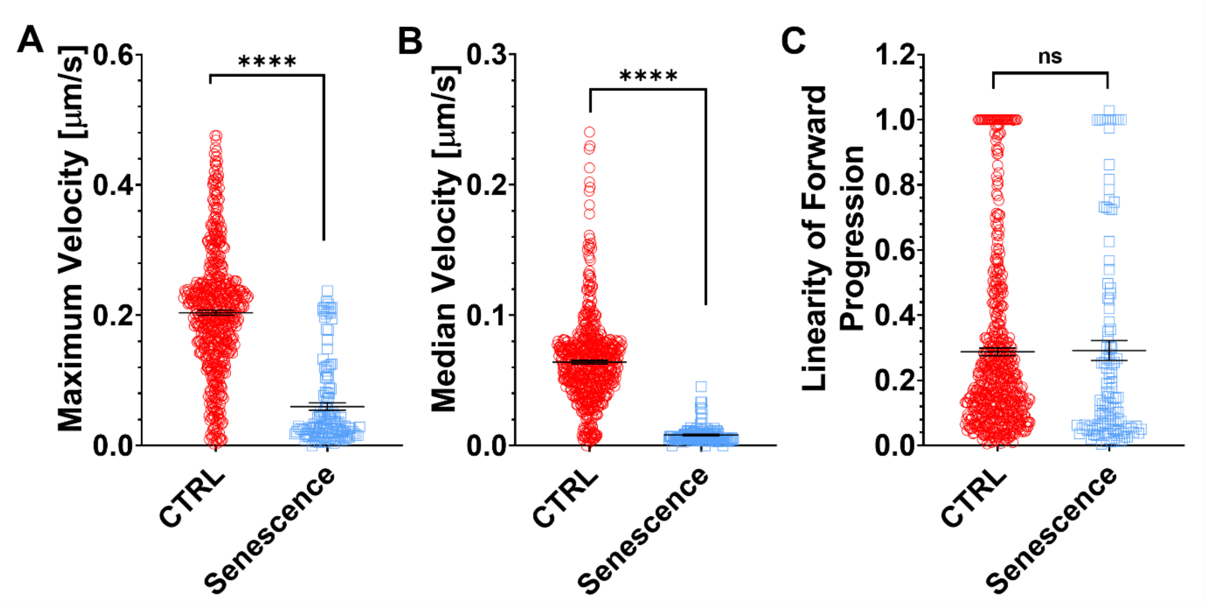

Supplementary Figure 5. Dynamics transport parameters of LDs in control and senescent cells: A) maximum velocity, B) median velocity, and C) linearity of forward progression (n=569 LDs for control cells and n=123 LDs for senescent cells). ns: not significant; ****, p<0.0001.

© 2024. Singam S. et al. Published online at http://www.aginganddisease.org/EN/10.14336/AD.2024.1408

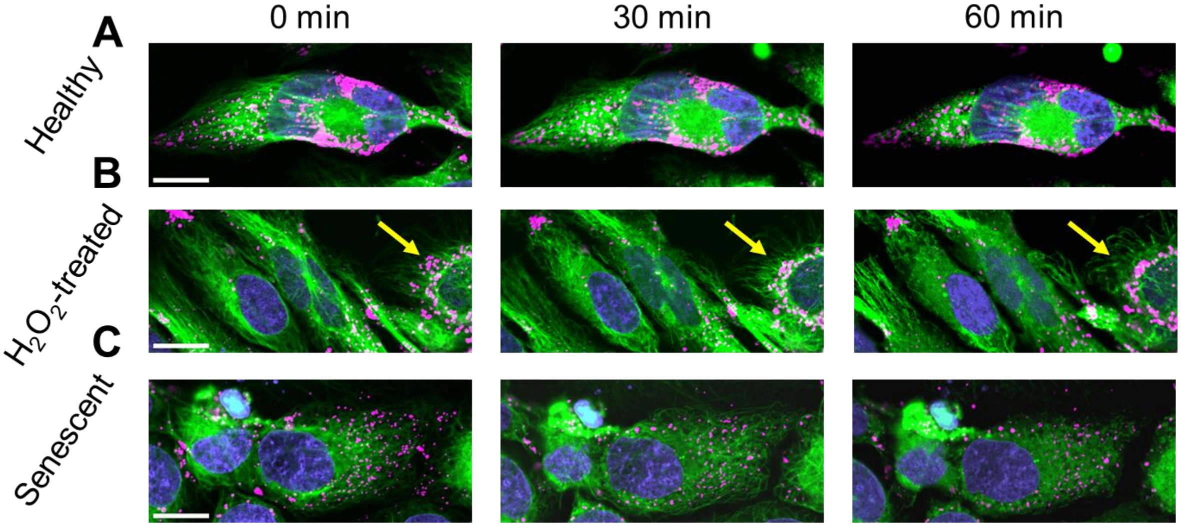

Supplementary Figure 6. LD movement on microtubules (MTs): In healthy cells, MTs are well-distributed and interconnected, facilitating the random motion of LDs (A). Shortly after H_2O_2 treatment, MTs become aligned toward the nucleus, driving the linear movement of LDs in that direction (B). In senescent cells, MT polymerization and intensity are significantly reduced, resulting in minimal LD movement due to the weakened MT network (C). The yellow arrow indicates the alignment of MTs and the corresponding linear movement of LDs along them. Green represents MTs, pink indicates LDs, and purple denotes the nucleus. Scale bar: 20 µm.

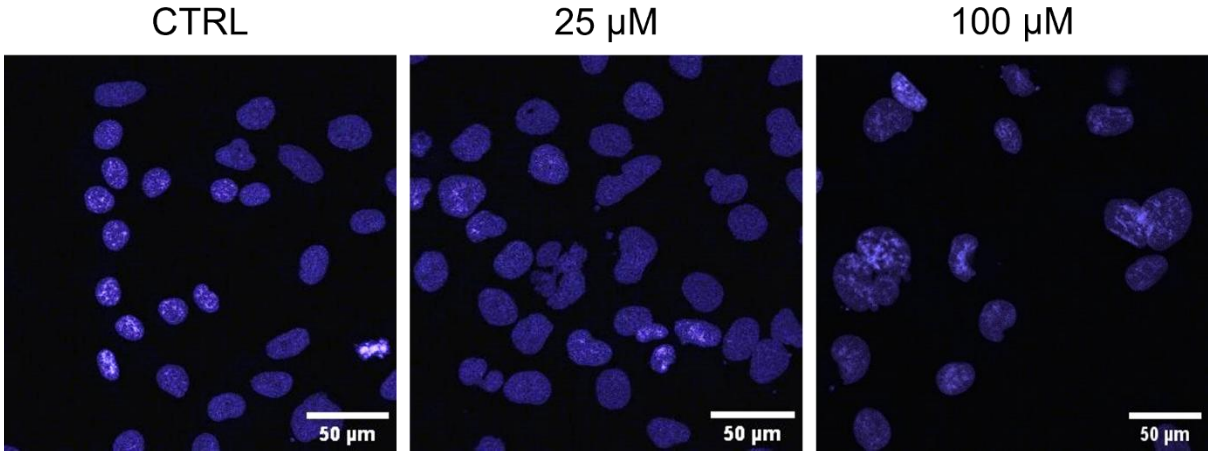

Supplementary Figure 7. Representative nuclear images of control cells and H_2O_2-induced senescent cells (25 µM and 100 µM).

© 2024. Singam S. et al. Published online at http://www.aginganddisease.org/EN/10.14336/AD.2024.1408

SUPPLEMENTARY DATA

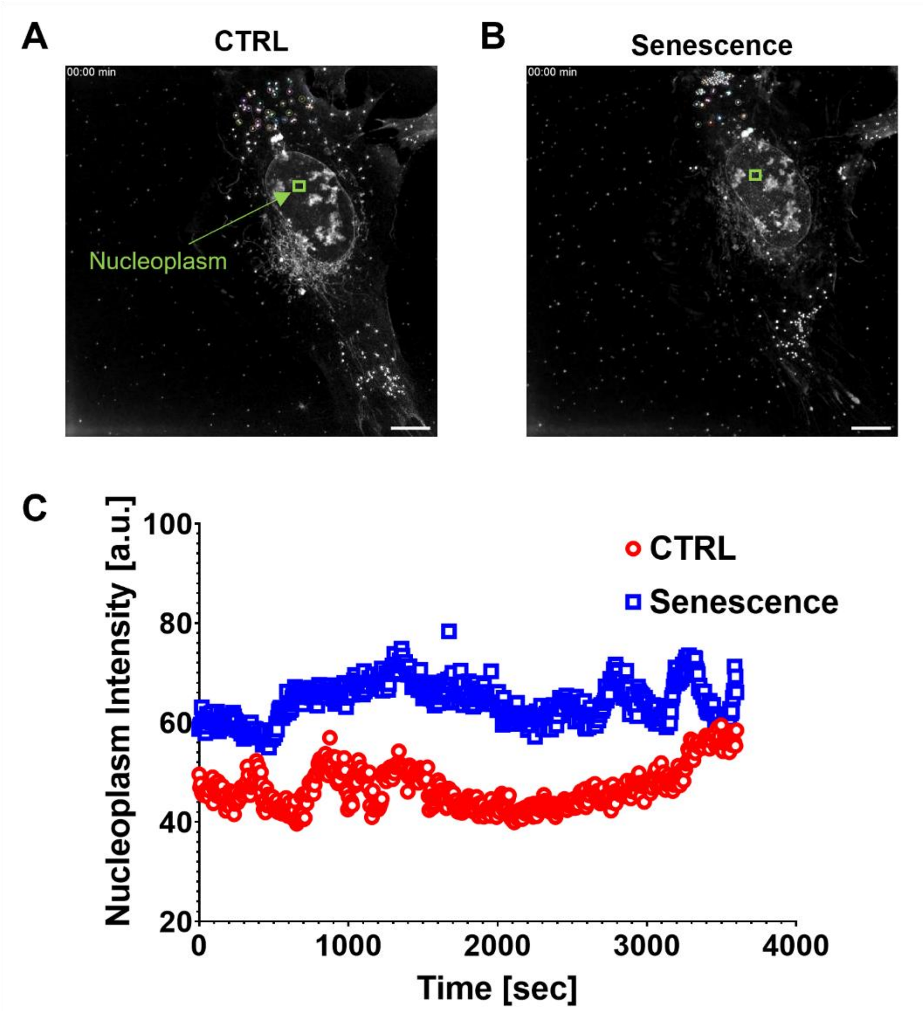

Supplementary Figure 8. Nucleoplasm intensity of control and senescent cells. Holotomographic images of control A) and senescent cells B). Scale bar: 10 µm. The average nucleoplasm intensity over time C) was calculated within the green rectangle.

© 2024. Singam S. et al. Published online at http://www.aginganddisease.org/EN/10.14336/AD.2024.1408

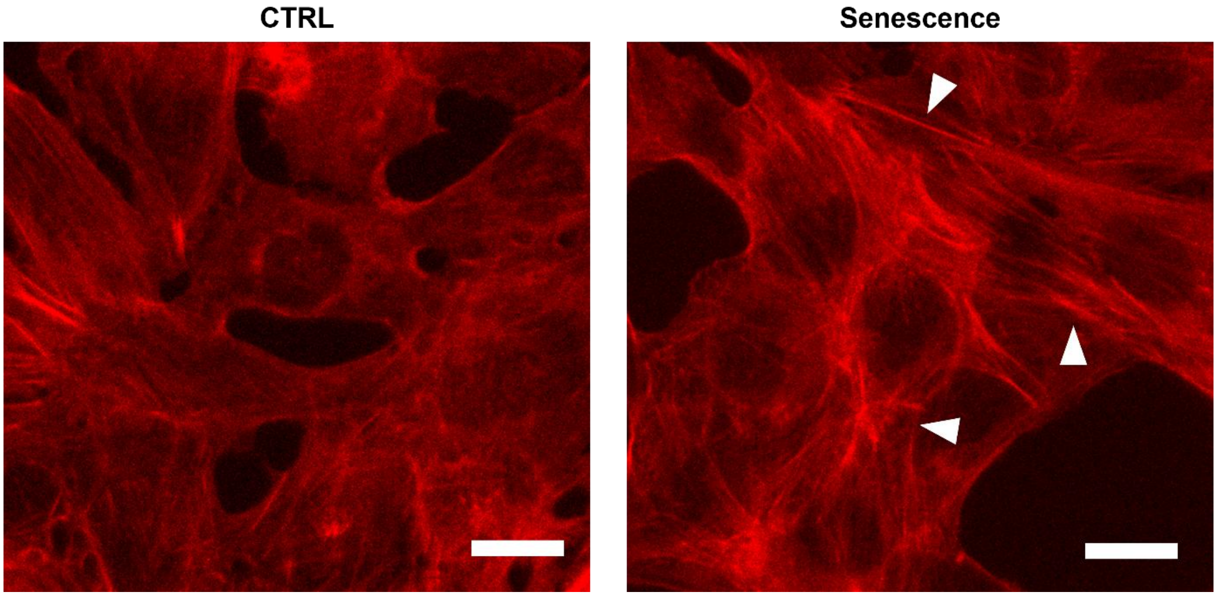

Supplementary Figure 9. Fluorescence images of the F-actin cytoskeleton in control and senescent cells (100 μM). White triangles indicate stress fibers. Scale bar: 20 μm.

© 2024. Singam S. et al. Published online at http://www.aginganddisease.org/EN/10.14336/AD.2024.1408
